# Pomegranate Juice Supplementation Alters Utero-Placental Vascular Function and Fetal Growth in the eNOS^−/−^ Mouse Model of Fetal Growth Restriction

**DOI:** 10.3389/fphys.2018.01145

**Published:** 2018-08-14

**Authors:** Sarah L. Finn-Sell, Elizabeth C. Cottrell, Susan L. Greenwood, Mark R. Dilworth, Elizabeth J. Cowley, Colin P. Sibley, Mark Wareing

**Affiliations:** ^1^Maternal and Fetal Health Research Centre, Division of Developmental Biology and Medicine, Faculty of Biology, Medicine and Health, University of Manchester, Manchester, United Kingdom; ^2^Saint Mary’s Hospital, Manchester University NHS Foundation Trust, Manchester Academic Health Science Centre, Manchester, United Kingdom

**Keywords:** pomegranate, vascular function, FGR, pregnancy, mouse

## Abstract

The eNOS^−/−^ mouse provides a well-characterized model of fetal growth restriction (FGR) with altered uterine and umbilical artery function and reduced utero- and feto-placental blood flow. Pomegranate juice (PJ), which is rich in antioxidants and bioactive polyphenols, has been posited as a beneficial dietary supplement to promote cardiovascular health. We hypothesized that maternal supplementation with PJ will improve uterine and umbilical artery function and thereby enhance fetal growth in the eNOS^−/−^ mouse model of FGR. Wild type (WT, C57Bl/6J) and eNOS^−/−^ mice were supplemented from E12.5-18.5 with either PJ in their drinking water or water alone. At E18.5 uterine (UtA) and umbilical (UmbA) arteries were isolated for study of vascular function, fetuses and placentas were weighed and fetal biometric measurements taken. PJ supplementation significantly increased UtA basal tone (both genotypes) and enhanced phenylephrine-induced contraction in eNOS^−/−^ but not WT mice. Conversely PJ significantly reduced UtA relaxation in response to both acetylcholine (Ach) and sodium nitroprusside (SNP), endothelium dependent and independent vasodilators respectively from WT but not eNOS^−/−^ mice. UmbA sensitivity to U46619-mediated contraction was increased by PJ supplementation in WT mice; PJ enhanced contraction and relaxation of UmbA to Ach and SNP respectively in both genotypes. Contrary to our hypothesis, the changes in artery function induced by PJ were not associated with an increase in fetal weight. However, PJ supplementation reduced litter size and fetal abdominal and head circumference in both genotypes. Collectively the data do not support maternal PJ supplementation as a safe or effective treatment for FGR.

## Introduction

Fetal growth restriction (FGR), where the fetus fails to reach its genetic growth potential, is a prevalent clinical problem affecting 3–5% of pregnancies (Miller et al., 2008; Bamfo and Odibo, 2011). FGR is defined as an estimated fetal weight of less than the 3rd centile or a contribution of two of the following factors: EFW less than the 10th centile, a 2 quartile drop in growth centiles, or abnormal uterine artery Doppler pulsatility index greater than the 95th centile, (Gordijn et al., 2016). FGR is associated with both short and long-term adverse outcomes, increasing the risk of perinatal morbidity and mortality (Bernstein et al., 2000; Ødegard et al., 2000; Bukowski et al., 2014; Chaiworapongsa et al., 2014), as well as the development of cardio-metabolic disease in later life (Barker, 2006). Despite the severe consequences of FGR, there remain limited interventions available to clinicians to either improve pregnancy outcome (e.g., low dose aspirin and steroid administration) or to mitigate the adverse outcomes for FGR neonates (Alberry and Soothill, 2007; Sibley, 2017). There is a clear need for the development of safe and effective interventions to improve pregnancy outcomes.

The underlying etiology of FGR is complex but placental dysfunction underlies most idiopathic cases (Cartwright et al., 2010). This may arise from inadequate spiral artery remodeling (Harris, 2010), an adaptation of early pregnancy which lowers vascular resistance and facilitates maternal blood flow to the placenta (Burton et al., 2009; Cartwright et al., 2010). Failed artery remodeling leads to placental hypoxia/oxidative stress and abnormal development of the feto-placental blood vessels (Burton and Jauniaux, 2011). Severe early onset FGR (before 34 weeks gestation), is associated with abnormal umbilical artery and uterine artery flow-velocity as determined clinically by Doppler waveform analysis (Ghosh and Gudmundsson, 2009). Abnormal vascular development and regulation of vascular tone both contribute to inadequate blood flow from mother to placenta, and between placenta and fetus, which compromises fetal growth and development (Jones et al., 2015).

The endothelial nitric oxide synthase knock out (eNOS^−/−^) mouse provides a well-characterized model of FGR, with phenotypic similarities to human FGR where; fetuses are ∼10% smaller compared with their wild type (WT) counterparts near term (Kusinski et al., 2012; Kulandavelu et al., 2013), and knockout animals demonstrate impaired uterine artery reactivity (Kusinski et al., 2012), reduced uterine and umbilical blood flow (Kulandavelu et al., 2012) and increased systolic blood pressure (Shesely et al., 1996). Additionally, in common with human FGR, there is evidence of increased reactive oxygen species (ROS) generation in the placentas of eNOS^−/−^ mice (Kusinski et al., 2012) compared to WT. Whilst in pregnancy there is an increase in ROS production due to the high metabolic rate of the placenta (Myatt and Cui, 2004), excessive ROS production or a deficit in cellular anti-oxidant mechanisms can result in oxidative damage within the placenta and its vasculature (Weseler and Bast, 2010); this oxidative damage is thought to contribute to many pregnancy complications including FGR (Burton and Jauniaux, 2004).

Pomegranate juice (PJ) consumption has been demonstrated to have a number of beneficial cardiovascular effects (Aviram and Rosenblat, 2012) and is often advocated as a well tolerated dietary supplement to improve vascular health (Basu and Penugonda, 2009). These beneficial actions of PJ are thought to be due to the high concentration of bioactive polyphenol compounds including: anthocyanins, tannins (particularly the ellagitannin, punicalagin), phenols and flavonoids, found in pomegranate as well as other fruits (Stoclet et al., 2004; McCutcheon et al., 2008). Indeed isolated phenolic compounds and extracts of fruits, including pomegranate, have been show to exhibit important anti-oxidant actions, and to enhance isolated vessel reactivity in rats (Fitzpatrick et al., 1993; Ajay et al., 2003; Delgado et al., 2017). Furthermore pomegranate has the ability to reduce systolic blood pressure in both a rat model of hypertension (Delgado et al., 2017) and in hypertensive patients (Aviram and Dornfeld, 2001). In pregnancy, PJ supplementation has been shown to limit oxidative stress induced injury, both *in vitro* (reducing oxidative stress and apoptosis in placental trophoblast cells) and *in vivo* (following maternal consumption) in the placentas of pre-eclamptic patients (Chen et al., 2012, 2013).

Given the promising effects of pomegranate polyphenols on vascular function in non-pregnant animal models and humans, the ability of PJ to promote utero- and fetoplacental vascular function during fetal development and potentially improve fetal growth in FGR is worthy of investigation. Furthermore, despite the beneficial effects of pomegranate consumptions on adult health, little is known about the effect(s) of high polyphenol consumption on fetal development (Ly et al., 2015) and it is likely that the developing fetus may be particularly susceptible to perturbations in redox balance (Ufer et al., 2010).

Here, we tested the hypothesis that maternal PJ supplementation would improve uterine and umbilical artery function and increase fetal growth in the eNOS^−/−^ model of FGR, without any gross perturbations to fetal development.

## Materials and Methods

### Mice and Ethical Approval

Animal care and experimental procedures were performed in accordance with the UK Animals (Scientific Procedures) Act 1986 under Home Office licenses PPL 40/3385 and PPL 70/8504. The Local Ethical Review Process of the University of Manchester approved all protocols. eNOS^−/−^ mice were obtained from Jackson Laboratories (strainB6.129P2-Nos3tm1Unc/J). C57/BL6J mice, the background strain, were used as the WT control. Mice were mated and the presence of a copulation plug was denoted as embryonic day (E) 0.5 of pregnancy. All female mice were 10–16 weeks old at time of mating; male mice were 12–26 weeks old. At E12.5 (term E19.5) mice were randomly assigned to receive either PJ administered via the drinking water or drinking water alone for the remainder of pregnancy. E12.5 was chosen as the time-point of intervention to coincide with a gestational age analogous to human second trimester when FGR could reasonably be diagnosed and therapeutic intervention initiated. Animals had free access to food (BK001 diet, Special Dietary Services, United Kingdom) and water (Hydropac, Lab products Inc, Seaford, DE, United States) or PJ diluted in water (see specific dose information below), and were maintained on a 12:12-h light-dark cycle at 21–23°C in individually ventilated cages. All animals were sacrificed by cervical dislocation at E18.5 and the placentas, fetuses, uterine and umbilical arteries harvested.

### Pomegranate Juice Treatment

Pomegranate juice treated dams received a PJ solution of commercially available POM wonderful juice [Los Angeles, CA, United States (McCutcheon et al., 2008)] diluted in drinking water to a final concentration of 3% (v/v). This provides an expected dose of 0.31 μmol polyphenols/day or 0.55 mg/kg, based on an expected fluid consumption of 5 ml per mouse per day, determined in previous studies in our group, (Dilworth et al., 2013). This dose was in line with doses previously shown to have biological activity in animal models (Aviram et al., 2000) and was further chosen with the aim of providing animals with an equivalent dose to a human adult drinking a 240 ml (8oz) glass of juice daily, equivalent to many clinical trial doses (McCutcheon et al., 2008; Chen et al., 2012). Drinking bottles contained 100 ml of PJ solution or water and were changed each morning (8am – 10am) and drinking volumes calculated based on fluid remaining. Fluid intake (E12.5 – E18.5) was comparable for all treatments and maternal weight gain was not different between genotype or treatment groups (WT = 10.13 g ± 7.6, eNOS^−/−^ = 8.57 g ± 1.05, WT+PJ = 8.9 g ± 3.37, eNOS^−/−^ +PJ = 8.5 g ± 1.46). Median baseline weights at the commencement of treatment was 27 grams, (range 23.5–29.5 g).

### Fetal and Placental Measurements

Fetal and placental wet weights were measured at E18.5 and growth curves constructed as previously described (Dilworth et al., 2011). Biometric measurements (crown-rump length, abdominal circumference and head circumference) were made as previously described (Dilworth et al., 2011).

### Wire Myography

Main loop uterine arteries (UtAs) and umbilical arteries (UmbAs) were dissected from eNOS^−/−^ and WT dams and fetuses at E18.5 and mounted onto a wire myograph (Danish Myo Technologies 610M; DMT, Aarhus, Denmark) and bathed in physiological salt solution (PSS) as described previously (Kusinski et al., 2009). Normalization procedures were performed as described in detail previously (Wareing et al., 2002), whereby artery diameters were normalized to 0.9L_13.3_ for UtAs and 0.9L_5.1_ for UmbAs (diameter of vessel at which luminal pressures were 13.3 and 5.1 kPa respectively); calculated vessel diameters were recorded. Vessels were allowed to equilibrate for 20 min post normalization before the commencement of vasoactive studies. Maximal smooth muscle contraction was measured by exposing vessels to KPSS solution (120 mM KCl substituted for NaCl). Drug-induced contraction was measured using incremental doses of phenylephrine (PE;10^−9^ to 10^−5^M) in uterine arteries and thromboxane mimetic U46619 (10^−9^ – 2 × 10^−6^M) in umbilical arteries at 2 min intervals. Endothelium-dependent relaxation to acetylcholine (Ach; 10^−9^ to 10^−5^M) and endothelium-independent relaxation to sodium nitro-prusside (SNP; 10^−9^ to 10^−5^M) was assessed in UtAs and UmbAs pre-contracted with 10^−5^M PE and an EC_80_ of U46619 (as calculated from the U46619 dose response curve), respectively.

### Data Analysis

For myography analysis, average responses from 2 to 4 vessels (UmbA) or 2–4 vessel segments (UtA) per dam were calculated from *N* = 14–20 dams (UtA) and *N* = 11–13 dams (UmbA), and were compared between genotype and treatment groups. Artery contraction is expressed as active effective pressure (kPa) calculated as follows:

$$\begin{array}{r} {\mathrm{Active effective pressure K}}_{\text{i}}(\text{kPa})=\mathrm{Vessel tension}(\text{nM}/{\text{mm}}^{2})/\\ \left[\mathrm{Vessel diameter}(\mu )*\;2000\right] \end{array}$$

Artery relaxation is presented as active effective pressure in response to the vasodilator expressed as a % of the maximum pre-constriction achieved with PE or U46619 (later denoted as 100%). Data are presented as mean ± SEM with *n* = number of vessels, *N* = number of animals. Statistical analysis is via two-way ANOVA, with Sidak’s *post hoc* test where appropriate. EC_50_ concentrations for dose-response curves were calculated for each vessel individually by applying a sigmoidal dose response curve fit to the data. Differences in vessel diameter, basal tone, KPSS response and EC50s were assessed by two-way ANOVA or Kruskal–Wallis test and data are presented as individual data points with median. Sample size calculations were based on a 5% significance level, with 80% statistical power. For an approximate 15% improvement in vascular function (based on increase in maximal Ach-induced relaxation), with a standard deviation of 18%, effect size estimate is 0.850. A minimum sample size of 11 animals per group is therefore required.

Litter means for placental weight, fetal weight were calculated and the data are presented as scatterplots with median of *N* = 14–20 l. Fetal weight distribution curves are also presented. Statistical analysis was via two-way ANOVA or Kruskal–Wallis test as appropriate.

## Results

### Effect of PJ Supplementation on Uterine Artery Reactivity

**FIGURE 1:**
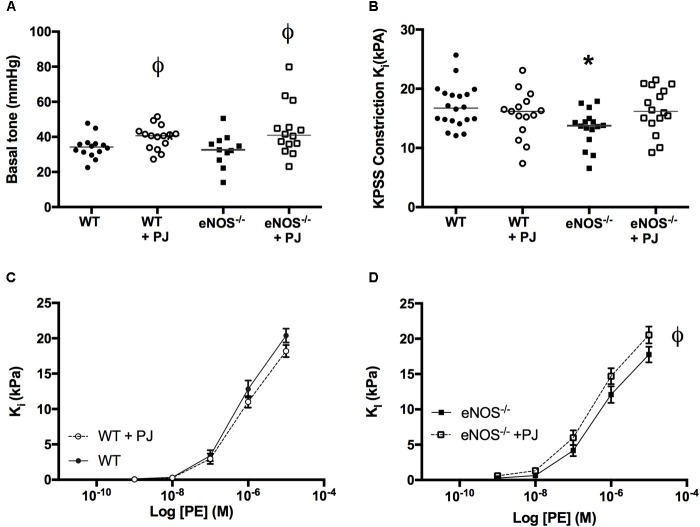
Contraction of uterine arteries. Pomegranate juice significantly increased uterine artery (UtA) basal tone vs. untreated vessels irrespective of genotype **(A)**. The initial constriction to KPSS **(B)** was lower in eNOS^−/−^ mice compared to WT. Data are mean ± SEM of 2–4 vessels/animal *N* = 14–20 dams/group, lines represent median. ^ϕ^*P* < 0.05, significant effect of treatment vs. genotype matched control, one-way ANOVA. ^∗^*P* < 0.05 significant effect of genotype. PJ supplementation did not affect uterine artery contraction to phenylephrine in WT mice **(C)** but significantly enhanced contraction in eNOS^−/−^ mice **(D)**. Data are mean ± SEM ^ϕ^*P* < 0.05, significant effect of treatment, two-way ANOVA.

UtAs of eNOS^−/−^ mice were ∼15–20% smaller in diameter than arteries of WT mice irrespective of treatment (WT = 254 μm ± 7, eNOS^−/−^ = 212 μm ± 9, WT + PJ = 237 μm ± 7, eNOS^−/−^ +PJ = 203 μm ± 9, two-way ANOVA effect of genotype, *P* < 0.0001). Basal tone of normalized vessels (i.e., tone at passive relaxation), expressed as mmHg, was not affected by genotype but was increased in vessels from both WT and eNOS^−/−^ mice that had been consuming PJ (**Figure 1A**, two-way ANOVA significant effect of treatment). The initial contraction to KPSS was lower in eNOS^−/−^ mice (vs. WT), but this effect was not evident in vessels from PJ supplemented eNOS^−/−^ mice. PJ supplementation had no effect on KPSS-induced contraction in arteries from WT mice (**Figure 1B**). UtA contraction in response to PE was similar in eNOS^−/−^ and WT mice, but in eNOS^−/−^ mice, PJ supplementation significantly increased contraction to PE vs. water controls (**Figures 1C,D**).

**FIGURE 2:**
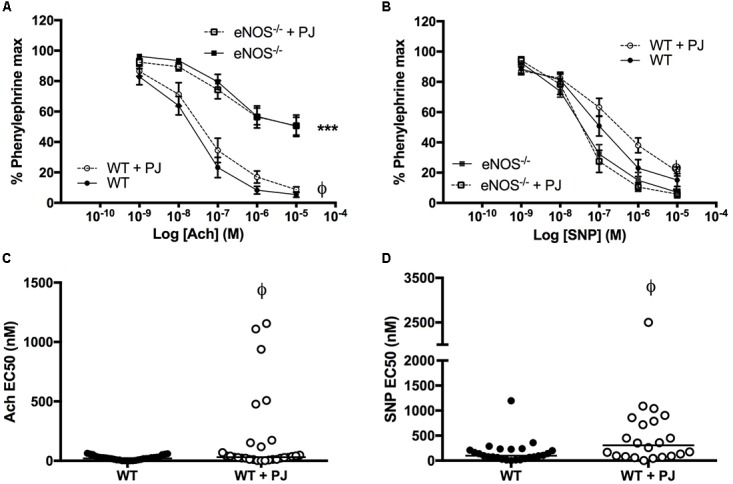
Relaxation of uterine arteries. Relaxation of UtAs from WT and eNOS^−/−^ mice in response to Acetlycholine (Ach) **(A)** and Sodium Nitroprusside (SNP) **(B)**. Relaxation in response to Ach was significantly reduced, whereas SNP induced relaxation was significantly enhanced in eNOS^−/−^ mice compared to WT irrespective of treatment. Pomegranate juice supplementation significantly reduced the relaxation responses to both Ach and SNP in WT but not eNOS^−/−^ animals. Data are mean ± SEM of averages of 2–4 vessels/animal *N* = 14–20 dams/group. EC_50_ doses of both Ach **(C)** and SNP **(D)** were higher in WT animals supplemented with PJ representing a decreased sensitivity of UTAs following supplementation. *n* = 22–31 vessels, line = median. ^∗^*P* < O.05, ^∗∗∗^*P* < 0.001, significant effect of genotype. ^ϕ^*P* < 0.05, significant effect of treatment.

Consistent with the eNOS^−/−^ phenotype reported by us (Kusinski et al., 2012) and others (Kulandavelu et al., 2012, 2013), relaxation to Ach was significantly attenuated and relaxation to SNP was potentiated in eNOS^−/−^ compared with WT mice, (**Figures 2A,B**). PJ supplementation reduced agonist induced UtA relaxation to both Ach and SNP in WT but not eNOS^−/−^ mice. This reduced relaxation response was paralleled by a reduced sensitivity to agonists with a significant increase in EC_50_ (**Figures 2C,D**) in WT mice. There was no effect of PJ supplementation on EC_50_ for Ach and SNP-induced relaxation in eNOS^−/−^ mice (data not shown).

### Effect of PJ Supplementation on Umbilical Artery Reactivity

As with UtAs, UmbAs from eNOS^−/−^ mice were smaller than WT (WT = 482 μm ± 12, eNOS^−/−^ = 445 μm ± 12, two-way ANOVA effect of genotype, *p* = 0.0112) and there was no effect of PJ supplementation on vessel diameter. There was no effect of genotype or treatment on basal tone or KPSS contraction (data not shown).

**FIGURE 3:**
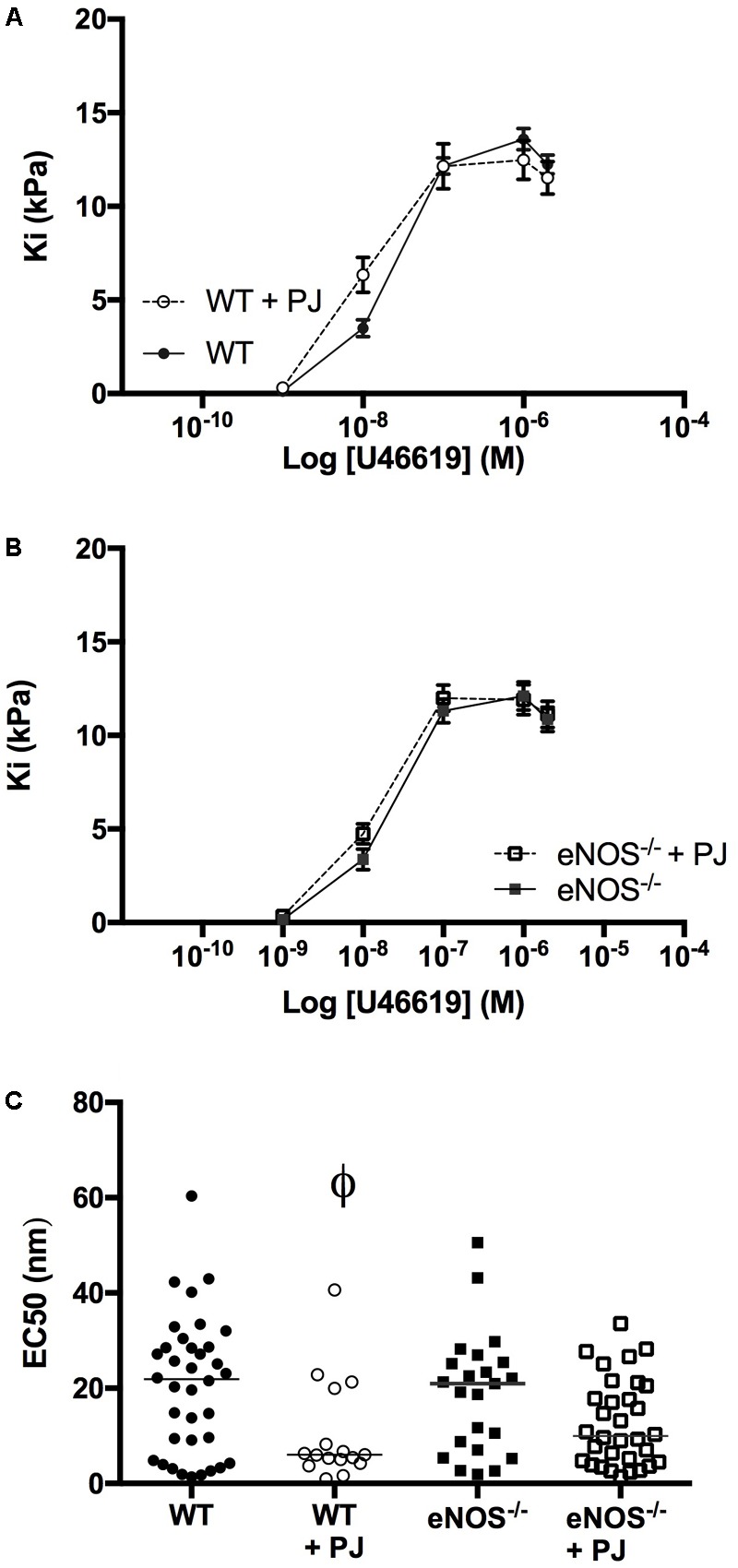
Umbilical artery contraction. PJ supplementation did not affect the maximal UmbA contraction in response to U46619 in either WT **(A)** or eNOS^−/−^ **(B)** mice, *N* = 11–13 dams, *n* = 28–36 vessels. The EC_50_ dose **(C)** was significantly lower in vessels from WT mice supplemented with PJ. ^ϕ^*P* < 0.05, significant effect of treatment in WT vessels, two-way ANOVA.

UmbA maximal contraction to U46619 was not affected by PJ supplementation in either WT or eNOS^−/−^ mice (**Figures 3A,B**); however the sensitivity to U46619 was increased following PJ supplementation, with a significant reduction in EC_50_ in vessels from WT animals (**Figure 3C**).

**FIGURE 4:**
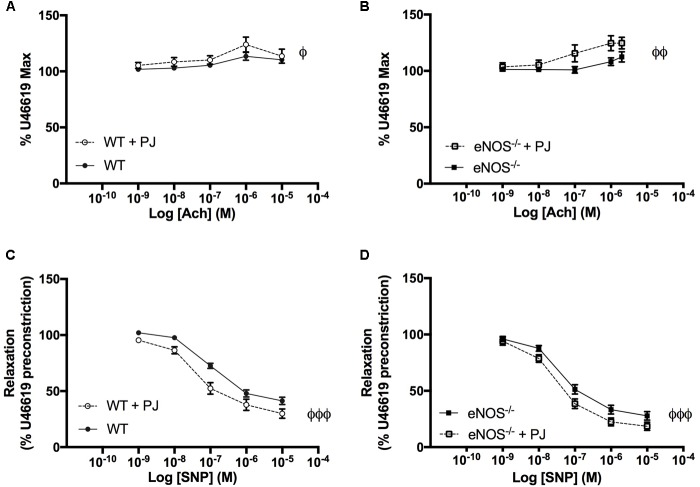
Effect of ACh and SNP on umbilical arteries. Ach induced contraction of UmbAs from both WT **(A)** and eNOS^−/−^**(B)** mice. Contraction was increased following PJ supplementation in both genotypes. SNP induced relaxation of UmbAs in WT **(C)** and eNOS^−/−^**(D)** mice and relaxation was potentiated by PJ supplementation in both genotypes. *N* = 11–13 dams, *n* = 28–36 vessels. ^ϕ^*P* < 0.05, ^ϕϕ^*P* < 0.01, ^ϕϕϕ^*P* < 0.001 significant effect of treatment two-way ANOVA.

As previously reported UmbAs contracted in response to increasing concentrations of Ach (Kusinski et al., 2009), an effect that was significantly increased following PJ supplementation in both WT and eNOS^−/−^ mice (**Figures 4A,B**). In addition, relaxation to SNP was potentiated by PJ treatment in UmbAs of both WT and eNOS^−/−^ mice (**Figures 4C,D**).

### Effect of PJ Supplementation on Fetal Characteristics

**FIGURE 5:**
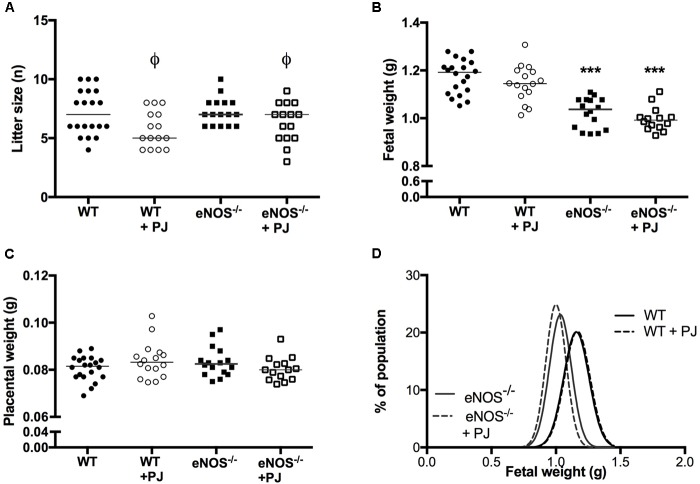
Litter size, fetal and placental weight. Litter size **(A)** was significantly reduced by PJ treatment, in both eNOS^−/−^ and WT mice (^ϕ^*P* < 0.05, two-way ANOVA). Fetal weight was lower in eNOS^−/−^ mice irrespective of treatment **(B)**, ^∗∗∗^*p* < 0.001, two-way ANOVA. There was no effect of genotype or treatment on placental weight **(C)**. *N* = 14–20 l. Fetal weight distribution curves **(D)** showed no differences in weight distribution following treatment, *n* = 78–143 pups from *N* = 14–20 l.

Litter size of both WT and eNOS^−/−^ mice was significantly reduced by PJ treatment (**Figure 5A**). However there was no corresponding effect on number of fetal resorptions (data not shown). eNOS^−/−^ fetuses were smaller than their WT counterparts (**Figures 5B,D**); neither mean fetal weight not fetal weight distribution was altered by PJ treatment in either genotype irrespective of fetal sex (data not shown). Placental weight was comparable between all groups (**Figure 5C**) resulting in a significantly decreased fetal:placental weight ratio in eNOS^−/−^ litters (WT = 14.02 ± 0.22, eNOS^−/−^ = 12.41 ± 0.23, *p* < 0.001). Consistent with their growth restricted phenotype, eNOS^−/−^ fetuses were smaller than their WT counterparts, with the largest differences seen in abdominal circumference suggesting a degree of head sparing (**Table 1**). Despite the lack of an effect of PJ on fetal weight, biometric measurements were affected by PJ supplementation (**Table 1**). These effects tended to be detrimental with significant reductions in both abdominal circumference and head circumference in WT and eNOS^−/−^ mice treated with PJ.

**Table 1:** Biometric measurements.

Biometric measurements	WT	WT + PJ	eNOS^−/−^	eNOS^−/−^ + PJ
Crown-rump length (mm)	29 (27–31)	28 (28–31)	28 (26–29)^∗^	28 (27–29)^∗^
Head circumference (mm)	29 (26–29)	27 (27–28)^ϕ^	28 (25–29)^∗^	26 (24–29)^∗ϕ^
Abdominal circumference (mm)	27 (24–29)	27 (25–27)^ϕ^	26 (24–28)^∗∗^	25 (23–27)^∗∗ϕ^

*Measurements of crown-rump length, head circumference and abdominal circumference median (range), N = 14–20 l, n = 56–80 fetuses. eNOS^−/−^ fetus’ were smaller by all measures compared to WT (two-way ANOVA: ^∗^p < O.05, ^∗∗^p < 0.01 vs. WT control). PJ supplementation significantly reduced head circumference and abdominal circumference irrespective of genotype (two-way ANOVA, ^ϕ^P < 0.05 vs. genotype matched water drinking control).*

## Discussion

Contrary to our hypothesis, PJ supplementation from E12.5-E18.5 of pregnancy did not improve fetal growth in eNOS^−/−^ mice. However it did have pronounced effects on vascular function of both UtAs and UmbAs with interesting differential effects in the different vascular beds and in different genotypes. In WT animals, PJ supplementation decreased sensitivity to agonist-induced relaxation (Ach and SNP) in UtAs whereas in UmbAs relaxation responses to SNP were increased, with PJ also increasing sensitivity of UmbAs to contraction in response to both U46619 and Ach. In eNOS^−/−^ animals, PJ supplementation had the same effects on UmbAs (enhancing contractile responses and increasing relaxation to SNP) but in UtAs contraction to PE was enhanced with no observable effect on relaxation responses. These findings, along with the significant reduction in litter size, and the reduction in fetal head and abdominal circumference irrespective of genotype, do not support maternal PJ supplementation as an effective therapy to improve fetal growth in FGR.

### Differential Effects of Pomegranate Juice Supplementation on Vasculature in Different Genotypes

In eNOS^−/−^ mice, UtA and UmbA diameters were smaller but basal tone was comparable. Vessel reactivity of UtAs in untreated eNOS^−/−^ mice was significantly decreased, with reduced artery contraction to depolarizing concentrations of potassium and blunted endothelial dependent relaxation to Ach, as demonstrated previously (Chataigneau et al., 1999; Kusinski et al., 2012). Relaxation to the endothelial independent agonist (SNP) was potentiated in eNOS^−/−^ mice, in common with previous observations (Brandes et al., 2000), thought to be attributed to an increased sensitivity to soluble guanylyl cyclase. Overall these data support previous observations of a reduced vaso-reactivity of UtAs of eNOS^−/−^ mice and a reduced uterine blood flow (Kulandavelu et al., 2012), although the latter was not measured in this study. The differential effects of PJ on UtAs in the two genotypes may provide clues as to the mechanisms of action of PJ in the uterine vasculature and suggest that, unsurprisingly, differential antioxidant pathways predominate in the absence of the eNOS enzyme.

The structural properties of the phenolic constituents of PJ can mediate scavenging of a number of radicals, including superoxide, hydroxyl groups and peroxy-nitrite, (McCutcheon et al., 2008) and (in WT) potentiate NO bioavailability (Gryglewski et al., 1987). This can complement endogenous oxidative stress defense mechanisms to improve vascular health (Stoclet et al., 2004; Burton and Jauniaux, 2011), mitigating the damaging effects of these radicals. In the vasculature this has been shown to improve vasodilator responses by preventing the radical mediated oxidation of LDL, which is known to impair endothelium dependent relaxation(Deckert et al., 1997). Polyphenols also augment NO-independent components of vascular relaxation including enhancing Endothelial Derived Hyperpolarizing Factor (EDHF) actions (Vanhoutte, 2004) and stimulating smooth muscle relaxation following voltage-gated ion channel activation (Ndiaye et al., 2003), both of which can increase the production of the vasodilator prostacyclin (Mizugaki et al., 2000) and decrease production of the constrictor endothelin-1 (Corder et al., 2001). Furthermore in WT animals polyphenols interact with eNOS-cGMP signaling (Fitzpatrick et al., 1993; Stoclet et al., 2004) and with antioxidants, increasing eNOS enzyme expression (Ramasamy et al., 1999) and NO production (Andriambeloson et al., 1997).

Despite this wealth of evidence supporting positive effects of pomegranate polyphenols on vascular health, PJ treatment did not beneficially affect vascular function in either WT or eNOS^−/−^ mice in the current study. In eNOS^−/−^ mice, PJ led not to enhanced relaxation but enhanced constriction. Any beneficial effects of PJ on components of vascular reactivity appears insufficient to counteract the deficit in vasodilator effects of NO in the absence of endogenous eNOS in the uterine vasculature of eNOS^−/−^ animals. In WT animals, vessel reactivity to exogenous vasodilators was blunted by PJ treatment in contrast to the positive effects seen in other models, (Fitzpatrick et al., 1993; Ajay et al., 2003; Delgado et al., 2017). However, the enhanced constriction to PE observed in UtAs of eNOS^−/−^ mice was absent in arteries from WT mice, again suggesting that the presence of eNOS may provide a protective role above and beyond the actions of PJ.

### Effects of Pomegranate Juice in Umbilical Vessels

It has been demonstrated previously that UmbAs of WT mice do not demonstrate a consistent contractile response to PE (Kusinski et al., 2009) and so the thromboxane mimetic U46619 was used to compare the effect of PJ treatment on UmbA contraction in WT and eNOS^−/−^ mice. Furthermore, UmbAs, in contrast to most arteries including UtAs, contract rather than relax in response to Ach (Kusinski et al., 2009). Agonist induced responses of UmbAs following PJ supplementation were similar between the genotypes suggesting that eNOS and nitric oxide may play a less prominent role in the relaxation of these vessels. Additionally, agonist induced responses were enhanced for all compounds under investigation, both contractile and vasodilatory, either by enhancing sensitivity or maximal responses. Interestingly, although in the majority of vascular beds prostaglandins mediate artery relaxation, in umbilical vessels prostaglandins trigger constriction (Abramovich et al., 1984). This may help explain the increased umbilical artery sensitivity to U46619 following PJ treatment as polyphenols have been demonstrated to increase prostacyclin (PGI_2_) production by endothelial cells in culture (Mizugaki et al., 2000). A tendency for UmbAs to constrict may reduce blood flow to the developing fetus, negatively impacting nutrient delivery and fetal growth.

A further consideration is the bioavailability of the polyphenol constituents in PJ and their metabolites not only in the systemic circulation but also the placenta, (Unadkat et al., 2004). Although these data are supportive of dietary polyphenols being present in the placenta and fetal circulation, the milieu of polyphenols present in the fetal circulation is likely to be quite distinct from that in maternal plasma and this could contribute to differences in the effects of PJ on opposing sides of the maternal-fetal interface.

### Excessive Polyphenol Consumption May Be Detrimental to Developmental Outcomes

The observed effects on vascular function of both uterine and umbilical vessels were not associated with any improvement in fetal weight in eNOS^−/−^ and there were modest reductions in biometric measurements following PJ supplementation. Polyphenols have previously been shown to negatively affect both fetal development, by causing premature closure of the ductus arteriosus in the third trimester (Zielinsky and Busato, 2013), and nutrient transport processes (reviewed by Martel et al., 2010). Interestingly, different polyphenols can have differential effects on nutrient transport. For example, the polyphenols rutin, catechin and quercetin, which are all present in PJ (McCutcheon et al., 2008), have been shown to differentially affect glucose transport in placental cells (Araújo et al., 2008). We were unable to make an assessment of the bioavailability of polyphenols in the present study due to small sample volumes available, a limitation of many preclinical studies. Nevertheless, such alterations in transport could potentially contribute to the growth suppressing effects of PJ supplementation in the current study.

A recently published paper, (Chen et al., 2018), examined effect of PJ supplementation in a hypoxic model (12% O_2_ vs. 21% O_2_) of FGR. PJ had no effect on fetal or placental weight in control animals but was able to improve fetal weight under conditions of hypoxia compared to water drinking controls. However, the PJ concentration used was three times that in the present study and the enhancement in fetal weight was in part due to a restoration of calories to these animals; glucose supplementation used to control for energy intake in this study also improved fetal weight.

Monsefi et al. (2012) reported that embryo femur length increased following treatment of mice with PJ extract (an in-house prepared PJ extract, administered at 3.3 ml/kg between days 8 and 18 of pregnancy), and that PJ increased cell proliferation and differentiation *in vitro*, consistent with a positive effect of PJ on bone cells. However, a bone growth-promoting effect of PJ (administered from E12.5) was not evident in the current study, as PJ did not affect crown rump length. It is possible that crown-rump length might have increased with extended PJ supplementation; however, the effect of PJ to reduce head circumference and abdominal circumference observed in the current study does not support maternal treatment with PJ over a longer term in our model.

In addition to the lack of improvement in fetal weight in eNOS^−/−^ mice, a striking observation in the present study was a reduction in litter size in both genotypes following PJ supplementation. Interestingly, one of the mechanisms whereby PJ can reduce blood pressure in hypertensive patients is via decreasing angiotensin converting enzyme (ACE) activity (Aviram and Dornfeld, 2001). Whist the use of ACE inhibitor drugs are common and are well tolerated in non-pregnant adults, the safety of these drugs in pregnant women has come under question in recent years with other classes of anti-hypertensives being recommended in this patient sub-group (Magee et al., 1999). Specifically the use of ACE inhibitors or angiotensin II receptor blockers in pregnancy can lead to low birth weight as well as an increased miscarriage rate compared to untreated pregnant women (Moretti et al., 2012). This may be a mechanism underpinning the reduction in litter size following PJ supplementation in the current study.

The period of PJ supplementation in the present study can be considered to be roughly equivalent to the second and third trimesters of human pregnancy. The timing of treatment was chosen as it is from the equivalent point in human pregnancy that babies are identified as growth restricted and an intervention could realistically be introduced. However, in the mouse this time also coincides with significant placental growth to E16.5 followed by rapid fetal growth and development (Coan et al., 2004). Although beyond the scope of this study, differences in placental function with PJ supplementation during this critical window of development may have adversely effected fetal development as observed in other studies (Sferruzzi-Perri and Camm, 2016).

Our results indicate that pomegranate supplementation may be detrimental to fetal outcomes in this eNOS^−/−^ mouse model of FGR, which results from of a single gene deletion. Whilst the eNOS^−/−^ model demonstrates many phenotypic similarities to human FGR it is important to note that the etiology of human FGR is complex and our results should be interpreted with caution and investigation in alternative mouse models is warranted before extrapolating the results to pregnant women.

## Conclusion

Whilst in other models pomegranate supplementation can have highly beneficial effects on multiple measures of cardiovascular health and vascular function these improvements were not observed in the current study. Indeed, maternal PJ supplementation was associated with a range of detrimental effects on utero-and fetoplacental vascular function, litter size and fetal growth. Although elevated oxidative stress has been implicated in pregnancy complications such as FGR and preeclampsia, artificially altering the anti-oxidant milieu during pregnancy may not always be beneficial (Poston et al., 2006). If the effects of PJ on vascular function and fetal well-being observed here were mediated through its actions as an anti-oxidant, our results indicate that upsetting the balance between the generation and sequestration of reactive oxygen species could result in poor pregnancy outcomes. Collectively the data do not support maternal PJ supplementation as a safe or effective treatment for FGR.

## Author Contributions

SG, CS, and MW formulated the research question. SF-S, ECC, and SG designed the study. SF-S, ECC, MD, EJC, and MW performed the research. SF-S, ECC, and SG analyzed the data. SF-S wrote the manuscript and all authors approved the final version.

## Conflict of Interest Statement

The authors declare that the research was conducted in the absence of any commercial or financial relationships that could be construed as a potential conflict of interest.

## References

[B1] Abramovich D. R. Page K. R. Parkin A. M. (1984). The effect of prostaglandin D2 on the blood vessels of the perfused isolated cotyledon of the human placenta. *Br. J. Pharmacol.* 81 19–21. 10.1111/j.1476-5381.1984.tb10737.x 6584189 PMC1986948

[B2] Ajay M. Gilani A.-U. H. Mustafa M. R. (2003). Effects of flavonoids on vascular smooth muscle of the isolated rat thoracic aorta. *Life Sci.* 74 603–612. 10.1016/j.lfs.2003.06.039 14623031

[B3] Alberry M. Soothill P. (2007). Management of fetal growth restriction. *Arch. Dis. Child. Fetal Neonatal Ed.* 92 F62–F67. 10.1136/adc.2005.082297 17185432 PMC2675309

[B4] Andriambeloson E. Kleschyov A. L. Muller B. Beretz A. Stoclet J. C. Andriantsitohaina R. (1997). Nitric oxide production and endothelium-dependent vasorelaxation induced by wine polyphenols in rat aorta. *Br. J. Pharmacol.* 120 1053–1058. 10.1038/sj.bjp.0701011 9134217 PMC1564573

[B5] Araújo J. R. Gonçalves P. Martel F. (2008). Modulation of glucose uptake in a human choriocarcinoma cell line (BeWo) by dietary bioactive compounds and drugs of abuse. *J. Biochem.* 144 177–186. 10.1093/jb/mvn054 18424810

[B6] Aviram M. Dornfeld L. (2001). Pomegranate juice consumption inhibits serum angiotensin converting enzyme activity and reduces systolic blood pressure. *Atherosclerosis* 158 195–198. 10.1016/S0021-9150(01)00412-9 11500191

[B7] Aviram M. Dornfeld L. Rosenblat M. Volkova N. Kaplan M. Coleman R. (2000). Pomegranate juice consumption reduces oxidative stress, atherogenic modifications to LDL, and platelet aggregation: studies in humans and in atherosclerotic apolipoprotein E-deficient mice. *Am. J. Clin. Nutr.* 71 1062–1076. 10.1093/ajcn/71.5.1062 10799367

[B8] Aviram M. Rosenblat M. (2012). Pomegranate protection against cardiovascular diseases. *Evid. Based Complement. Altern. Med.* 2012:382763. 10.1155/2012/382763 23243442 PMC3514854

[B9] Bamfo J. E. A. K. Odibo A. O. (2011). Diagnosis and management of fetal growth restriction. *J. Pregnancy* 2011:640715. 10.1155/2011/640715 21547092 PMC3087156

[B10] Barker D. J. (2006). Adult consequences of fetal growth restriction. *Clin. Obstet. Gynecol.* 49 270–283. 10.1097/00003081-200606000-00009 16721106

[B11] Basu A. Penugonda K. (2009). Pomegranate juice: a heart-healthy fruit juice. *Nutr. Rev.* 67 49–56. 10.1111/j.1753-4887.2008.00133.x 19146506

[B12] Bernstein I. M. Horbar J. D. Badger G. J. Ohlsson A. Golan A. (2000). Morbidity and mortality among very-low-birth-weight neonates with intrauterine growth restriction. *Am. J. Obstet. Gynecol.* 182 198–206. 10.1016/S0002-9378(00)70513-8 10649179

[B13] Brandes R. P. Kim D.-Y. Schmitz-Winnenthal F.-H. Amidi M. Gödecke A. Mülsch A. (2000). Increased nitrovasodilator sensitivity in endothelial nitric oxide synthase knockout mice. Role of soluble guanylyl cyclase. *Hypertension* 35 231–236. 10.1161/01.hyp.35.1.231 10642303

[B14] Bukowski R. Hansen N. I. Willinger M. Reddy U. M. Parker C. B. Pinar H. (2014). Fetal growth and risk of stillbirth: a population-based case–control study. *PLoS Med.* 11:e1001633. 10.1371/journal.pmed.1001633 24755550 PMC3995658

[B15] Burton G. J. Jauniaux E. (2004). Placental oxidative stress: from miscarriage to preeclampsia. *J. Soc. Gynecol. Investig.* 11 342–352. 10.1016/j.jsgi.2004.03.003 15350246

[B16] Burton G. J. Jauniaux E. (2011). Oxidative stress. *Best Pract. Res. Clin. Obstet. Gynaecol.* 25 287–299. 10.1016/j.bpobgyn.2010.10.016 21130690 PMC3101336

[B17] Burton G. J. Woods A. W. Jauniaux E. Kingdom J. C. P. (2009). Rheological and physiological consequences of conversion of the maternal spiral arteries for uteroplacental blood flow during human pregnancy. *Placenta* 30 473–482. 10.1016/j.placenta.2009.02.009 19375795 PMC2697319

[B18] Cartwright J. E. Fraser R. Leslie K. Wallace A. E. James J. L. (2010). Remodelling at the maternal–fetal interface: relevance to human pregnancy disorders. *Reproduction* 140 803–813. 10.1530/rep-10-0294 20837731

[B19] Chaiworapongsa T. Chaemsaithong P. Yeo L. Romero R. (2014). Pre-eclampsia part 1: current understanding of its pathophysiology. *Nat. Rev. Nephrol.* 10 466–480. 10.1038/nrneph.2014.102 25003615 PMC5893150

[B20] Chataigneau T. Félétou M. Huang P. L. Fishman M. C. Duhault J. Vanhoutte P. M. (1999). Acetylcholine-induced relaxation in blood vessels from endothelial nitric oxide synthase knockout mice. *Br. J. Pharmacol.* 126 219–226. 10.1038/sj.bjp.0702300 10051139 PMC1565804

[B21] Chen B. Longtine M. S. Nelson D. M. (2013). Punicalagin, a polyphenol in pomegranate juice, downregulates p53 and attenuates hypoxia-induced apoptosis in cultured human placental syncytiotrophoblasts. *Am. J. Physiol. Endocrinol. Metab.* 305 E1274–E1280. 10.1152/ajpendo.00218.2013 24085032 PMC3840214

[B22] Chen B. Longtine M. S. Riley J. K. Nelson D. M. (2018). Antenatal pomegranate juice rescues hypoxia-induced fetal growth restriction in pregnant mice while reducing placental cell stress and apoptosis. *Placenta* 66 1–7. 10.1016/j.placenta.2018.04.009 29884297

[B23] Chen B. Tuuli M. G. Longtine M. S. Shin J. S. Lawrence R. Inder T. (2012). Pomegranate juice and punicalagin attenuate oxidative stress and apoptosis in human placenta and in human placental trophoblasts. *Am. J. Physiol. Endocrinol. Metab.* 302 E1142–E1152. 10.1152/ajpendo.00003.2012 22374759 PMC3361977

[B24] Coan P. M. Ferguson-Smith A. C. Burton G. J. (2004). Developmental dynamics of the definitive mouse placenta assessed by stereology1. *Biol. Reprod.* 70 1806–1813. 10.1095/biolreprod.103.024166 14973263

[B25] Corder R. Douthwaite J. A. Lees D. M. Khan N. Q. Viseu Dos Santos A. C. Wood E. G. (2001). Endothelin-1 synthesis reduced by red wine. *Nature* 414 863–864. 10.1038/414863a 11780050

[B26] Deckert V. Persegol L. Viens L. Lizard G. Athias A. Lallemant C. (1997). Inhibitors of arterial relaxation among components of human oxidized low-density lipoproteins. Cholesterol derivatives oxidized in position 7 are potent inhibitors of endothelium-dependent relaxation. *Circulation* 95 723–731. 10.1161/01.CIR.95.3.723 9024163

[B27] Delgado N. T. B. Rouver W. D. N. Freitas-Lima L. C. de Paula T. D.-C. Duarte A. Silva J. F. (2017). Pomegranate extract enhances endothelium-dependent coronary relaxation in isolated perfused hearts from spontaneously hypertensive ovariectomized rats. *Front. Pharmacol.* 7:522. 10.3389/fphar.2016.00522 28101057 PMC5209391

[B28] Dilworth M. R. Andersson I. Renshall L. J. Cowley E. Baker P. Greenwood S. (2013). Sildenafil citrate increases fetal weight in a mouse model of fetal growth restriction with a normal vascular phenotype. *PLoS One* 8:e77748. 10.1371/journal.pone.0077748 24204949 PMC3813774

[B29] Dilworth M. R. Kusinski L. C. Baker B. C. Renshall L. J. Greenwood S. L. Sibley C. P. (2011). Defining fetal growth restriction in mice: a standardized and clinically relevant approach. *Placenta* 32 914–916. 10.1016/j.placenta.2011.08.007 21889207

[B30] Fitzpatrick D. F. Hirschfield S. L. Coffey R. G. (1993). Endothelium-dependent vasorelaxing activity of wine and other grape products. *Am. J. Physiol. Heart Circ. Physiol.* 265 H774–H778. 10.1152/ajpheart.1993.265.2.H774 8396352

[B31] Ghosh G. S. Gudmundsson S. (2009). Uterine and umbilical artery Doppler are comparable in predicting perinatal outcome of growth-restricted fetuses. *BJOG* 116 424–430. 10.1111/j.1471-0528.2008.02057.x 19187375

[B32] Gordijn S. J. Beune I. M. Thilaganathan B. Papageorghiou A. Baschat A. A. Baker P. N. (2016). Consensus definition of fetal growth restriction: a Delphi procedure. *Ultrasound Obstet. Gynecol.* 48 333–339. 10.1002/uog.15884 26909664

[B33] Gryglewski R. J. Korbut R. Robak J. Swies J. (1987). On the mechanism of antithrombotic action of flavonoids. *Biochem. Pharmacol.* 36 317–322. 10.1016/0006-2952(87)90288-7 3101704

[B34] Harris L. K. (2010). Review: trophoblast-vascular cell interactions in early pregnancy: how to remodel a vessel. *Placenta* 31(Suppl.), S93–S98. 10.1016/j.placenta.2009.12.012 20060584

[B35] Jones S. Bischof H. Lang I. Desoye G. Greenwood S. L. Johnstone E. D. (2015). Dysregulated flow-mediated vasodilatation in the human placenta in fetal growth restriction. *J. Physiol.* 593 3077–3092. 10.1113/JP270495 25920377 PMC4532528

[B36] Kulandavelu S. Whiteley K. J. Bainbridge S. A. Qu D. Adamson S. L. (2013). Endothelial NO synthase augments fetoplacental blood flow, placental vascularization, and fetal growth in mice. *Hypertension* 61 259–266. 10.1161/hypertensionaha.112.201996 23150513

[B37] Kulandavelu S. Whiteley K. J. Qu D. Mu J. Bainbridge S. A. Adamson S. L. (2012). Endothelial nitric oxide synthase deficiency reduces uterine blood flow, spiral artery elongation, and placental oxygenation in pregnant mice. *Hypertension* 60 231–238. 10.1161/HYPERTENSIONAHA.111.187559 22615111

[B38] Kusinski L. C. Baker P. N. Sibley C. P. Wareing M. (2009). In vitro assessment of mouse uterine and fetoplacental vascular function. *Reprod. Sci.* 16 740–748. 10.1177/1933719109336613 19443912

[B39] Kusinski L. C. Stanley J. L. Dilworth M. R. Hirt C. J. Andersson I. J. Renshall L. J. (2012). eNOS knockout mouse as a model of fetal growth restriction with an impaired uterine artery function and placental transport phenotype. *Am. J. Physiol. Regul. Integr. Comp. Physiol.* 303 R86–R93. 10.1152/ajpregu.00600.2011 22552791

[B40] Ly C. Yockell-Lelievre J. Ferraro Z. M. Arnason J. T. Ferrier J. Gruslin A. (2015). The effects of dietary polyphenols on reproductive health and early development. *Hum. Reprod. Update* 21 228–248. 10.1093/humupd/dmu058 25376587

[B41] Magee L. A. Ornstein M. P. von Dadelszen P. (1999). Management of hypertension in pregnancy. *BMJ* 318 1332–1336. 10.1136/bmj.318.7194.1332 10323823 PMC1115719

[B42] Martel F. Monteiro R. Calhau C. (2010). Effect of polyphenols on the intestinal and placental transport of some bioactive compounds. *Nutr. Res. Rev.* 23 47–64. 10.1017/S0954422410000053 20392307

[B43] McCutcheon A. Udani J. Brown D. (2008). *Scientific and Clinical Monograph for POM Wonderful Pomegranate Juice.* Austin, TX: American Botanical Council.

[B44] Miller J. Turan S. Baschat A. A. (2008). Fetal growth restriction. *Semin. Perinatol.* 32 274–280. 10.1053/j.semperi.2008.04.010 18652928

[B45] Mizugaki M. Ishizawa F. Yamazaki T. Hishinuma T. (2000). Epigallocatechin gallate increase the prostacyclin production of bovine aortic endothelial cells. *Prostaglandins Other Lipid Mediat.* 62 157–164. 10.1016/S0090-6980(00)00060-5 10938409

[B46] Monsefi M. Parvin F. Talaei-Khozani T. (2012). Effects of pomegranate extracts on cartilage, bone and mesenchymal cells of mouse fetuses. *Br. J. Nutr.* 107 683–690. 10.1017/S0007114511003394 21781378

[B47] Moretti M. E. Caprara D. Drehuta I. Yeung E. Cheung S. Federico L. (2012). The fetal safety of angiotensin converting enzyme inhibitors and angiotensin II receptor blockers. *Obstet. Gynecol. Int.* 2012:658310. 10.1155/2012/658310 22203847 PMC3238411

[B48] Myatt L. Cui X. (2004). Oxidative stress in the placenta. *Histochem. Cell Biol.* 122 369–382. 10.1007/s00418-004-0677-x 15248072

[B49] Ndiaye M. Chataigneau T. Andriantsitohaina R. Stoclet J. C. Schini-Kerth V. B. (2003). Red wine polyphenols cause endothelium-dependent EDHF-mediated relaxations in porcine coronary arteries via a redox-sensitive mechanism. *Biochem. Biophys. Res. Commun.* 310 371–377. 10.1016/j.bbrc.2003.09.028 14521920

[B50] Ødegard R. A. Vatten L. J. Nilsen S. T. Salvensen K. A. Austgulen R. (2000). Preeclampsia and Fetal Growth. *Obstet. Gynecol.* 96 950–955. 11084184

[B51] Poston L. Briley A. L. Seed P. T. Kelly F. J. Shennan A. H. Vitamins in Pre-eclampsia Trial Consortium. (2006). Vitamin C and vitamin E in pregnant women at risk for pre-eclampsia (VIP trial): randomised placebo-controlled trial. *Lancet* 367 1145–1154. 10.1016/S0140-6736(06)68433-X 16616557

[B52] Ramasamy S. Drummond G. R. Ahn J. Storek M. Pohl J. Parthasarathy S. (1999). Modulation of expression of endothelial nitric oxide synthase by nordihydroguaiaretic acid, a phenolic antioxidant in cultured endothelial cells. *Mol. Pharmacol.* 56 116–123. 10.1124/mol.56.1.116 10385691

[B53] Sferruzzi-Perri A. N. Camm E. J. (2016). The Programming power of the placenta. *Front. Physiol.* 7:33 10.3389/fphys.2016.00033 PMC4789467 27014074

[B54] Shesely E. G. Maeda N. Kim H.-S. Desai K. M. Krege J. H. Laubach V. E. (1996). Elevated blood pressures in mice lacking endothelial nitric oxide synthase. *Proc. Natl. Acad. Sci. U.S.A.* 93 13176–13181. 10.1073/pnas.93.23.13176 8917564 PMC24066

[B55] Sibley C. P. (2017). Treating the dysfunctional placenta. *J. Endocrinol.* 234 R81–R97. 10.1530/JOE-17-0185 28483805 PMC5516438

[B56] Stoclet J.-C. Chataigneau T. Ndiaye M. Oak M.-H. El Bedoui J. Chataigneau M. (2004). Vascular protection by dietary polyphenols. *Eur. J. Pharmacol.* 500 299–313. 10.1016/j.ejphar.2004.07.034 15464042

[B57] Ufer C. Wang C. C. Borchert A. Heydeck D. Kuhn H. (2010). Redox control in mammalian embryo development. *Antioxid. Redox Signal.* 13 833–875. 10.1089/ars.2009.3044 20367257

[B58] Unadkat J. D. Dahlin A. Vijay S. (2004). Placental drug transporters. *Curr. Drug Metab.* 5 125–131. 10.2174/1389200043489171 14965255

[B59] Vanhoutte P. M. (2004). Endothelium-dependent hyperpolarizations: the history. *Pharmacol. Res.* 49 503–508. 10.1016/j.phrs.2003.11.015 15026027

[B60] Wareing M. Crocker I. P. Warren A. Y. Taggart M. J. Baker P. N. (2002). Characterization of small arteries isolated from the human placental chorionic plate. *Placenta* 23 400–409. 10.1053/plac.2002.0825 12061856

[B61] Weseler A. R. Bast A. (2010). Oxidative stress and vascular function: implications for pharmacologic treatments. *Curr. Hypertens. Rep.* 12 154–161. 10.1007/s11906-010-0103-9 20424954 PMC2876260

[B62] Zielinsky P. Busato S. (2013). Prenatal effects of maternal consumption of polyphenol-rich foods in late pregnancy upon fetal ductus arteriosus. *Birth Defects Res.* 99 256–274. 10.1002/bdrc.21051 24339037 PMC4065350

